# Cardiac β2 adrenergic receptor deletion drives calmodulin kinase II upregulation to induce connective tissue growth factor in cardiac fibrosis and diastolic dysfunction

**DOI:** 10.1093/function/zqaf036

**Published:** 2025-08-12

**Authors:** Chaoqun Zhu, Meimi Zhao, Luqi Zhao, Mingfu Wu, Yang K Xiang

**Affiliations:** Department of Pharmacology, University of California at Davis, Davis, CA 95616, USA; Department of Pharmacology, University of California at Davis, Davis, CA 95616, USA; Department of Pharmaceutical Toxicology, China Medical University, Shenyang 110122, China; Department of Pharmacological and Pharmaceutical Sciences, University of Houston, Houston, TX 77204, USA; Department of Pharmacological and Pharmaceutical Sciences, University of Houston, Houston, TX 77204, USA; Department of Pharmacology, University of California at Davis, Davis, CA 95616, USA; VA Northern California Health Care System, Mather, CA 95655, USA

**Keywords:** β2-adrenergic receptor, calmodulin-dependent protein kinase II, L-type calcium channel blocker, fibrosis, connective tissue growth factor

## Abstract

Abnormalities of Ca^2+^ signaling in the heart lead to common cardiac remodeling in the pathogenesis of cardiovascular disorders. The activation of calmodulin-dependent protein kinase II (CaMKII) is regulated by elevated intracellular Ca^2+^ level in cardiomyocytes, driving the progression of myocardial dysfunction. In this study, using models of β_2_ adrenergic receptor (β_2_AR) deficiency in cardiomyocytes (β_2_AR-CKO), we observed an increased phosphorylation of CaMKII and upregulation of gene expression and protein level of the fibrotic marker connective tissue growth factor (CTGF) in the myocytes. *In vivo* treatment with the CaMKII inhibitor KN93 attenuated the upregulation of CTGF protein expression in β_2_AR-CKO hearts. Enhanced L-type calcium channel (LTCC) current was observed in β_2_AR-CKO cardiomyocytes following adrenergic stimulation, indicating a disruption of Ca^2+^ signaling. Treatment with the LTCC blocker nifedipine attenuated CaMKII activity and the expression of CTGF in β_2_AR-CKO hearts, confirming the upstream role of abnormal LTCC-Ca^2+^ signaling. Additionally, 8-month-old β_2_AR-CKO mice exhibited cardiac fibrosis and diastolic dysfunction. One month of *in vivo* nifedipine treatment improved both cardiac dysfunction and fibrosis in β_2_AR-CKO mice. These findings highlight the critical role of cardiomyocyte β_2_AR in maintaining LTCC-Ca^2+^ homeostasis. Loss of β_2_AR amplifies the Ca^2+^-CaMKII axis, promoting fibrosis and cardiomyopathy in aging hearts.

## Graphical Abstract

**Graphical Abstract:**
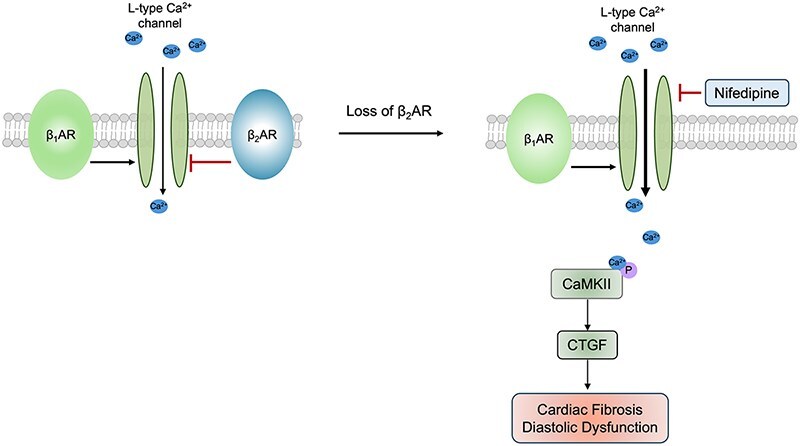
β2AR controls cardiac CTGF and fibrosis.

## Introduction

Calcium (Ca^2+^) homeostasis plays a pivotal role in maintaining normal cardiac function. Abnormalities of Ca^2+^ signaling in the heart lead to common cardiac remodeling in the pathogenesis of cardiovascular disorders.^1^ Ca^2+^/calmodulin-dependent protein kinase II (CaMKII) is a serine–threonine kinase and responds to changes in intracellular [Ca^2+^].^1^ While CaMKII activity is important for Ca^2+^ regulated physiological activities such as excitation–contraction coupling (ECC),^2^ excitation–transcription coupling (ETC),^3^ and “fight or flight” heart rate increases,^4^ it also contributes to arrhythmias, apoptosis, and gene expression favoring pathological remodeling in myocardial disease.^5,6^

β adrenergic receptors (βARs) regulate cardiac function in response to sympathetic stimulation. While β_1_AR is the predominant subtype in cardiomyocytes, responsible for most of the adrenergic-mediated cardiac contractility, β_2_AR has distinct signaling properties and plays a protective role in cardiomyocytes by regulating cellular survival pathways.^7^ Under the elevated sympathetic drive, chronic stimulation of β_1_AR induces CaMKII activity, which contributes to cardiac maladaptation and progression of heart failure.^8^ Cardiac β_2_AR has been suggested to blunt β_1_AR signaling by converting β_1_AR signaling from global to compartmentalized mode in dyads for physiological stress response and attenuate cardiac toxicity of chronic catecholamine insults.^9,10^

In this study, we investigated the interaction between cardiac β_2_AR signaling and Ca^2+^/CaMKII. Using cardiomyocyte-specific β_2_AR knockout mice, we found that β_2_AR deficiency leads to L-type calcium channel (LTCC)-dependent CaMKII activation in the heart, contributing to fibrosis and cardiac diastolic dysfunction in an age-dependent manner. Inhibition of CaMKII reduced expression of the pro-fibrotic factor connective tissue growth factor (CTGF) and the LTCC blocker nifedipine effectively mitigated the progression of cardiac fibrosis and dysfunction in the β_2_AR-deficient mice.

## Materials and Methods

### Animals

Age matched (2–8 months old) β_2_AR flox/flox (F/F), β_2_AR flox/flox with αMHC-Cre (cardiac knockout, CKO),^11^ and αMHC-Cre mice (JAX:009 074) were used in this study. All experiments, including echocardiogram, tissue harvesting, in vivo injection, and cardiomyocyte isolation, were approved by the Institutional Animal Care and Use Committees of the University of California, Davis, and followed NIH and ARRIVE guidelines. All experimental animals were housed at room temperature with a 12–12 h light cycle. Mice were anaesthetized with isoflurane (2%–5%) in oxygen through a nose cone during echocardiography and surgery. Mice were humanely euthanized for tissue harvest and cell isolation under deep isoflurane anesthesia. Hearts were quickly excised and snap frozen in liquid nitrogen and then transferred to a −80°C freezer for long-term storage. Alternatively, hearts were quickly rinsed in a chilled perfusion buffer for cannulation to isolate myocytes.

### Echocardiography

Mice were anaesthetized with isoflurane (1%–2%) in oxygen and placed on a warm pad with electrocardiogram monitoring. The heart rate was kept consistent between 450 and 550 beats per minute to avoid variation. Left ventricle systolic and diastolic functions were assessed using M-mode, tissue doppler, color doppler and pulsed wave doppler echocardiography using a Vevo 2100 Imaging System with a 22-25 MHz MS550D linear probe (Visual Sonic, Toronto, Canada).

### *In Vivo* CaMKII Inhibitor KN93 Treatment

Two to three-month-old male mice were subjected to receive CaMKII inhibitor KN93 treatment. β_2_AR F/F mice were injected intraperitoneally with KN93 (3 mg/kg/day), and β_2_AR CKO mice were injected with KN93 or KN92 for 14 days. Heart tissues were collected for western blot analysis.

### *In Vivo* LTCC Blocker Nifedipine Treatment

Two to three-month-old male mice were subjected to receive calcium channel blocker treatment. β_2_AR CKO mice were injected intraperitoneally daily with vehicle or nifedipine (N7634, Sigma–Aldrich, St. Louis, MO, USA) (10 mg/kg/day), and β_2_AR F/F mice were injected with vehicle for 14 days. Heart tissues were collected for western blot analysis. Six-month-old male mice were treated with nifedipine for 1 month. β2AR-CKO mice received daily intraperitoneal injections of either vehicle or nifedipine, while β2AR-F/F mice were injected with vehicle. Cardiac function was assessed at the end of the treatment period, and heart tissues were collected for western blot analysis.

### Histology Staining

Mice were sacrificed with heart harvest after anesthesia with 2%–3% isoflurane. Mouse hearts were perfused with 30 mm potassium chloride in phosphate-buffered saline (PBS) to arrest the hearts in diastole. Hearts were then fixed in 10% neutral formalin, embedded in paraffin, and sectioned (3 μm thickness). Paraffin-embedded sections were deparaffinized, rehydrated, and then stained for Hematoxylin and Eosin and Sirius Red. Stained sections were visualized and captured by a BZ-X710 microscope (Keyence, Itasca, IL, USA).

### Western Blot

Heart tissues were homogenized in lysis buffer (50 mm Tris-HCl, pH7.4, 150 mm NaCl, 5 mm EDTA, 0.5% sodium deoxycholate, 0.1% SDS, 1% NP-40) supplemented with proteinase and phosphatase inhibitors (1 M NaF, 0.1 M Na3VO4, 1 mm Bestatin, 2 mm E-64, 10 mm Pepstatin A, 0.1 M PMSF, 1 M Benzamidine). Tissue debris was removed by centrifugation at 12000 xg for 10 min at 4°C. The supernatant was transferred to a new tube and mixed with SDS-loading buffer. Equal amounts of protein were resolved by sodium dodecyl sulfate–polyacrylamide gel electrophoresis (SDS-PAGE) and detected with phospho-T286-CaMKII (ab32678, Abcam, Cambridge, UK), CaMKII (sc-5306, SCBT, Dallas, TX, USA), CTGF (HPA031075, Sigma, St. Louis, MO, USA), phospho-LTCC (Ser1928, custom antibody, Abmart, Berkeley Heights, NJ, USA), phospho-LTCC (Thr1700, custom antibody, Abmart, Berkeley Heights, NJ, USA), phospho-Ser16-phospholamban (A010-12, Badrilla, Leeds, UK), phospho-T17-phospholamban (A010-13, Badrilla, Leeds, UK), phospholamban (A010-14, Badrilla, Leeds, UK), AKT (9272S, Cell Signaling, Danvers, MA, USA), phospho-Ser473-AKT (D9E, Cell Signaling, Danvers, MA, USA), phospho-Thr308-AKT (244F9, Cell Signaling, Danvers, MA, USA), cleaved-caspase 3 (9664, Cell signaling, Danvers, MA), β_1_AR (sc-568, SCBT, Dallas, TX, USA), GAPDH (MAB374, Millipore Sigma, Burlington, MA, USA) was used as the loading control. The IRDye 800 CW goat anti-rabbit IgG (Licor, Lincoln, NE, USA) and IRDye 680 RD goat anti-mouse IgG (Licor, Lincoln, NE, USA) secondary antibodies were used to reveal primary antibodies using ChemiDoc^TM^ MP Imaging System (Bio-Rad, Hercules, CA, USA). The optical density of bands was analyzed with Fiji (https://fiji.sc). The arbitrary unit was defined as the ratio of intensity of protein of interest over the intensity of a reference protein or as the intensity of phosphorylated proteins over intensity of total proteins as indicated.

### Quantitative Real-Time PCR

Total RNAs were extracted from frozen heart tissues using TRIzol reagent (15596026, Invitrogen, Waltham, MA, USA) according to the manufacture’s instruction. cDNA was synthesized from the total RNA of heart samples using the High-Capacity cDNA Reverse Transcription Kit (4374967, Applied Biosystems, Waltham, MA, USA). Quantitative real-time PCR (qRT-PCR) was performed with SYBR Select Master Mix (A25742, Applied Biosystems, Waltham, MA, USA) in triplicate for each sample using primers listed in Supplemental Table 1 at an annealing temperature of 60°C. All qRT-PCR data were analyzed using the Applied Biosystems Comparative CT Method (ΔΔCT). Gene expression analysis was normalized to GAPDH.

### Adult Ventricular Cardiomyocytes Isolation

Adult mice (2−3 months old) were used for ventricular myocyte isolation.^12^ Briefly, mice were anaesthetized with 3% isoflurane, and hearts were quickly excised and rinsed in a chilled perfusion buffer with the composition: 120 mm NaCl, 5.4 mm KCl, 1.2 mm MgSO4.7H2O, 1.2 mm NaH_2_PO4, 2 mm NaHCO_3_, 10 mm 2,3-Butanedione monoxime, 5 mm Taurine, and 5.6 mm glucose, pH 7.4. The aorta was cannulated, and the heart was hung in a Langendorff perfusion apparatus. When the perfusate was clear of blood, the perfusion buffer was changed to a digestion buffer supplemented with 0.5 mg/mL type 2 collagenase (Worthington Biochemical, Lakewood, NJ, USA) and 0.1 mg/mL protease (XIV), and 50 μM CaCl_2_ until the heart was judged to be adequately digested. The aorta and atria were removed, and the ventricles were sliced. Ventricular myocytes were then released by gentle agitation using a transfer pipette. Cells were resuspended and recovered in the perfusion buffer with a gradient of Ca^2+^ to 1 mmol/L.

### Whole Cell Patch-Clamp Electrophysiology

Whole cell calcium currents (*I_Ca_*) were recorded from freshly isolated ventricular myocytes (2–3 months old) as described previously^13^ before and after 1 μM isoproterenol (I6540, Sigma–Aldrich, St. Louis, MO, USA) stimulation. Freshly isolated ventricular myocytes were maintained and perfused with Tyrode’s solution. After successful conversion to the whole cell patch configuration, the external solution was replaced with a Na^+^ free solution containing 140 mm NMDG, 5 mm CsCl, 1 mm MgCl_2_, 2 mm CaCl_2_, 10 mm Glucose, and 10 mm HEPES (pH 7.4 adjust with HCL). The fire polished borosilicate glass pipettes with 1–3 MΩ resistance were filled with a Cs-based internal solution containing 87 mm Cs-aspartate, 20 mm CsCl, 5 mm MgATP, 1 mm MgCl2, 10 mm EGTA, and 10 mm HEPES (pH 7.2 adjusted with CsOH). Total *I_Ca_* was elicited using a stimulation protocol from a holding potential of −80 mV to test potentials ranging from −40 to +60 mV. An additional 100 ms voltage step to −40 mV immediately preceded the depolarization pulses to inactivate voltage-gated Na^+^ channels. The experiment was performed at room temperature (22–25°C) using an Axopatch 200B amplifier (Molecular Devices, Sunnyvale, CA, USA) and a Digidata 1550B (Molecular Devices). The data were acquired using Clampex 10.7 (Molecular Devices, San Jose, CA, USA) and analyzed by Clampfit software (Molecular Devices, San Jose, CA, USA).

### Statistical Analysis

Pooled data were represented as the mean ± SEM. Fully blinded analysis was performed with different persons carrying out the experiments and analysis, respectively. No samples or animals were excluded from analysis. Representative figures and images reflected the average levels of each experiment. Normality of the data was assessed using two-sided Kolmogorov–Smirnov test in GraphPad Prism 9 (GraphPad Inc., San Diego, CA, USA). All statistical tests were two-sided and evaluated at a significance of 0.05. Unpaired two-tailed Student’s *t*-test was utilized for comparisons of two-independent variables. One-way analysis of variance (ANOVA) was used to compare 3 or more groups followed by Tukey’s multiple comparison. Two-way ANOVA was utilized for comparisons of two variables.

## Results

### Cardiomyocyte-specific Deletion of β_2_AR Enhances the Phosphorylation of CaMKII But Does Not Affect Cardiac Function in Young Mice

**Figure 1.**
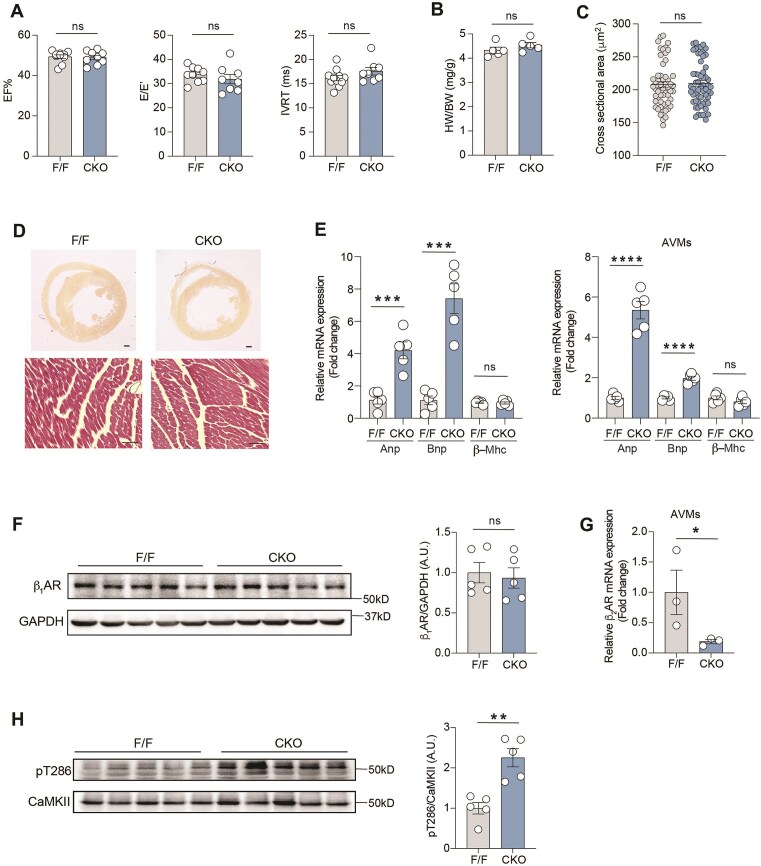
(A) Cardiac function in β_2_AR F/F and CKO mice as determined by ejection fraction (EF), the ratio of early diastolic transmitral blood flow velocity (E wave) to early diastolic mitral annular tissue velocity (E’ wave) (E/E’), isovolumic relaxation time (IVRT) (*n* = 8). (B) Heart weights of 2-month-old β_2_AR F/F and CKO mice were normalized to body weights (*n* = 5). (C) Quantification of cardiomyocyte cross sectional area (*n* = 50–60 cells from 5 animals) in 2-month-old β_2_AR F/F and CKO hearts. (D) Representative Sirius Red and Hematoxylin and Eosin stains of β_2_AR F/F and CKO heart cross sections at 2-month ages. Scale bar: 500 and 50 μm. (E) mRNA expression of hypertrophic markers Anp, Bnp, and β-Mhc in 2-month-old β_2_AR F/F and CKO hearts (*n* = 5) and isolated cardiomyocytes (*n* = 5 isolations). (F) Representative immunoblots and quantification of β_1_AR from β_2_AR F/F and CKO hearts (*n* = 5). (G) mRNA expression of *β_2_AR* in isolated cardiomyocytes (*n* = 3 isolations). (H) Representative immunoblots and quantifications of phosphorylated CaMKII and total CaMKII in β_2_AR F/F and CKO hearts (*n* = 5). Data are shown in dot plots with mean ± SEM. *P*-values were obtained by Student’s *t*-test. **P < 0.01; ***P < 0.001; ^****^P < 0.0001. ns, not significant.

We developed cardiomyocyte-specific β_2_AR knockout (β_2_AR-CKO) mice.^11^ β_2_AR deletion in the CKO heart was confirmed by significantly reduced β_2_AR mRNA expression in isolated adult ventricular cardiomyocytes (AVMs) (Figure 1G). At 2 months of age, β_2_AR-CKO mice had comparable systolic and diastolic functions to those in β_2_AR flox control (β_2_AR-F/F) mice (Figure 1A). Although heart weights (Figure 1B) and cardiac morphology (Figure 1C and D) remained unchanged in β_2_AR-CKO mice, there was a notable increase in the mRNA expression of cardiac hypertrophic markers atrial natriuretic peptide (ANP) and brain natriuretic peptide (BNP) in β_2_AR-CKO AVMs and hearts (Figure 1E), whereas expression of β-myosin heavy chain (β-MHC) was not altered (Figure 1E). To further explore the effect of β_2_AR deficiency on cardiac βAR signaling, we assessed the β_1_AR expression, which remained unaffected by the deletion of cardiomyocyte β_2_AR (Figure 1F). Interestingly, we observed a significant increase in CaMKII phosphorylation in β_2_AR-CKO hearts (Figure 1H).

### Elevation of CaMKII Activity Induces the Expression of Fibrotic Protein CTGF in β_2_AR-CKO Hearts

**Figure 2.**
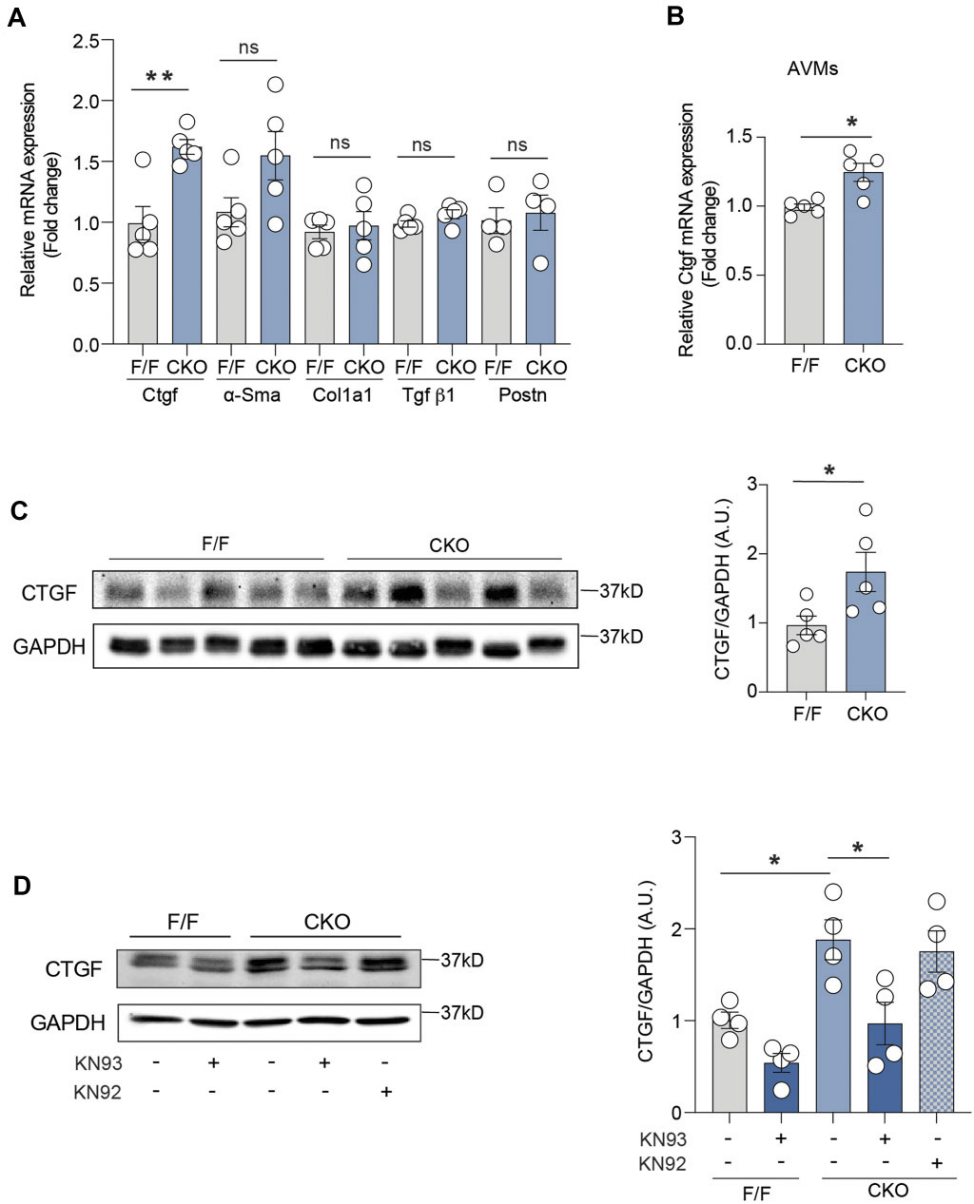
(A) Detection of mRNA expression of Ctgf, α-Sma, Col1a1, Tgf β1, and Postn in 2-month-old β_2_AR F/F and CKO hearts (*n* = 5). (B) Detection of mRNA expression of Ctgf in isolated cardiomyocytes from β_2_AR F/F and CKO hearts (*n* = 5 isolations). (C) Representative immunoblots and quantifications of CTGF in β_2_AR F/F and CKO hearts (*n* = 5). (D) Representative immunoblots and quantifications of CTGF in β_2_AR F/F and CKO hearts after in vivo 2-week of KN93 and KN92 treatment (*n* = 4). Dot plots represent the mean ± SEM. *P* values were obtained by Student’s *t*-test and one-way ANOVA. *P < 0.05; **P < 0.01. ns, not significant.

CaMKII plays central roles in intracellular Ca^2+^ signaling and processes maladaptive remodeling, inflammation, and fibrosis. We evaluated the expression of major inflammatory and fibrotic markers and found CTGF is regulated by the alteration of β_2_AR-CaMKII signaling cascade. The expression of Ctgf mRNA was upregulated in both β_2_AR-CKO heart tissues and AVMs (Figure 2A and B). The protein level of CTGF was also significantly increased in β_2_AR-CKO hearts (Figure 2C). To further confirm the effect of CaMKII activation on CTGF expression in the heart, we treated mice with CaMKII inhibitor, KN93, for 2 weeks in vivo. Treatment with KN93 led to attenuation of the expression level of CTGF in β_2_AR-CKO hearts (Figure 2D). KN92, an inactive analog of KN93, did not affect CTGF expression. These findings suggest that cardiac CTGF expression is regulated by CaMKII, with β_2_AR deficiency leading to CaMKII activation and subsequent upregulation of CTGF in the heart.

### Cardiomyocyte-specific Deletion of β_2_AR Enhances LTCC Current and Thereby Leads to CaMKII-CTGF Upregulation in the Heart

**Figure 3.**
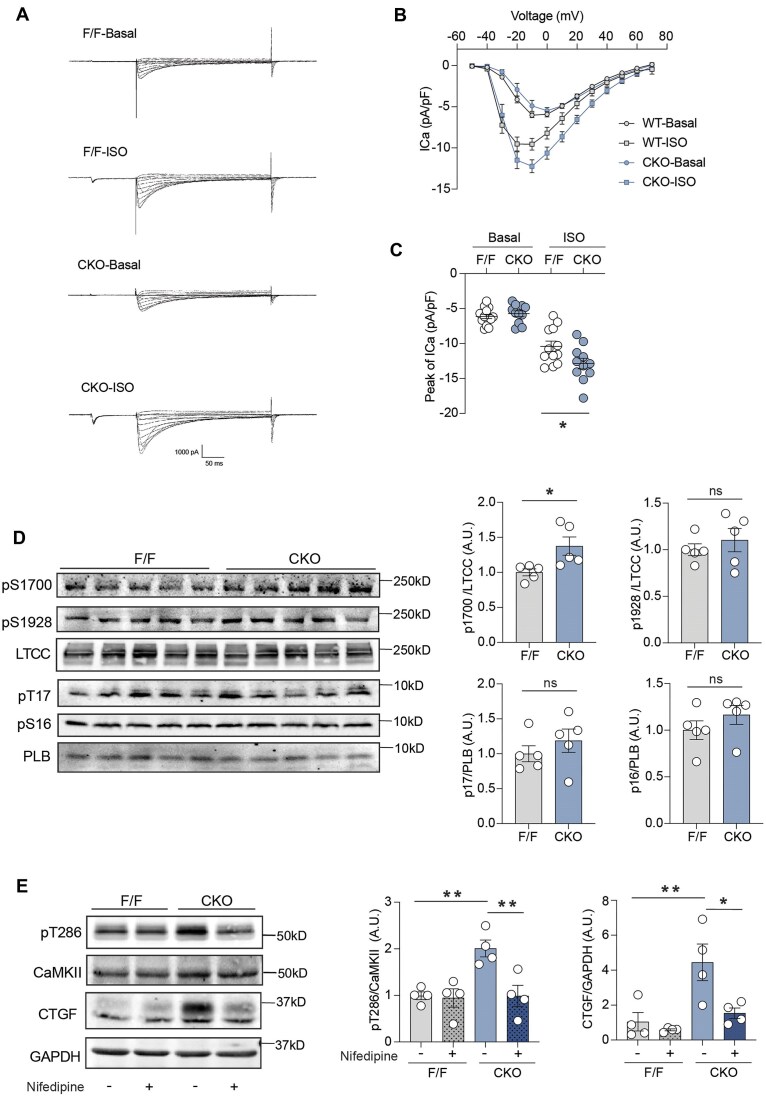
(A) *I_Ca_* current elicited from a representative 2-month-old β_2_AR F/F and CKO AVM before and during application of isoproterenol (ISO,1 μM). (B) *I*–*V* plot summarizes the *I_ca_* current results from multiple AVMs (*n* = 11–14 cells isolated from 7 mice) from β_2_AR F/F and CKO mice. (C) Peak currents are plotted (*n* = 11–14 cells isolated from 7 mice) from β_2_AR F/F and CKO mice. (D) Representative immunoblots and quantifications of phosphorylated total LTCC and phosphorylation of Serine 1700 and 1928, total PLB and phosphorylation of Serine 16 and Threonine 17 were detected in 2-month-old β_2_AR F/F and CKO hearts. (E) Representative immunoblots and quantifications of phosphorylated CaMKII, total CaMKII, and CTGF after in vivo 2-week of vehicle or nifedipine treatment (*n* = 4) in β_2_AR F/F and CKO mice. Dot plots represent the mean ± SEM. *P* values were obtained by one-way ANOVA. *P < 0.05; **P < 0.01.

CaMKII is activated by increases in intracellular [Ca^2+^]. LTCC is one of the major sources of increasing intracellular [Ca^2+^] in the cardiomyocyte. We hypothesized that loss of β_2_AR promotes LTCC activity in β_2_AR-CKO AVMs, leading to increased Ca^2+^ influx from the channel and consequently drives the activation of CaMKII. We performed whole-cell patch-clamp electrophysiology to record the βAR-stimulated whole-cell calcium current (*I_Ca_*) in freshly isolated AVMs. β_2_AR-CKO myocytes displayed a significantly higher *I_Ca_* than β_2_AR-F/F after stimulation of βAR with isoproterenol, while the baseline *I_Ca_* was similar between β_2_AR-F/F and β_2_AR-CKO AVMs (Figure 3A–C). Moreover, the phosphorylation of serine-1700 on LTCC was significantly upregulated (Figure 3D). In comparison, the phosphorylation of phospholamban (PLB) was not different between β_2_AR-CKO and β_2_AR-F/F hearts (Figure 3D). These findings support the notion that loss of β_2_AR disrupts compartmentalized signaling,^14,15^ leading to heightened LTCC phosphorylation and activity via unopposed β_1_AR–PKA signaling.

To evaluate the direct effect of LTCC on CaMKII activity and CTGF expression in β_2_AR-CKO hearts, we treated mice with a LTCC blocker, nifedipine. Two weeks of treatment with nifedipine abolished the increased phosphorylation of CaMKII and attenuated CTGF expression in β_2_AR-CKO hearts (Figure 3E). These results suggest that β_2_AR deficiency in cardiomyocytes leads to an increase in LTCC-Ca^2+^-CaMKII activity, inducing the expression of fibrotic protein CTGF in the heart.

### Elevation of Ca^2+^-CaMKII-CTGF Causes Cardiac Dysfunction and Remodeling in Aging β_2_AR-CKO Mice

**Figure 4.**
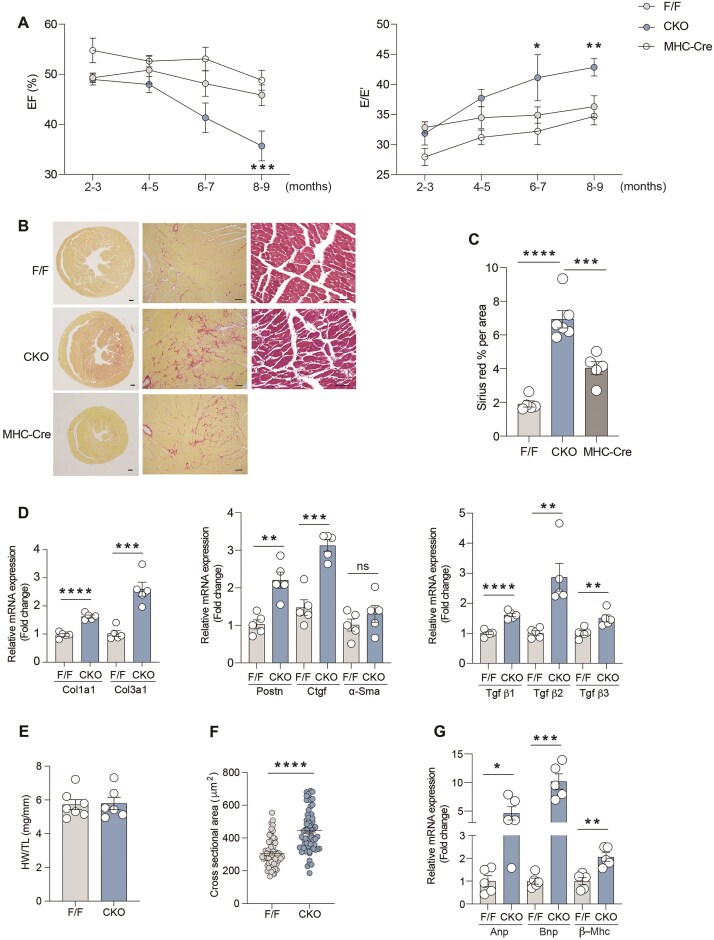
(A) Cardiac function was measured at different ages in β2AR F/F, β2AR CKO, and a-MHC Cre mice as determined by ejection fraction (EF) and the ratio of early diastolic transmitral blood flow velocity (E wave) to early diastolic mitral annular tissue velocity (E’ wave) (E/E’) (n = 7-9). (B) Representative Sirius Red and Hematoxylin and Eosin stains of β2AR F/F, β2AR CKO, and a-MHC Cre heart cross sections at 8-month ages (n = 5). Scale bar: 500 μm in whole heart section images and 50 μm in magnified images. (C) Quantification of fibrosis (%) by Sirius Red staining in β2AR F/F, β2AR CKO, and a-MHC Cre hearts (n =5). (D) Detection of mRNA expression of fibrotic makers Col1a1, Col3a1, Postn, Ctgf, a-Sma, Tgf β1, Tgf β2, Tgf β3 in 8-month-old β2AR F/F and CKO hearts (n = 5). (E) Heart weights of 8-month β2AR F/F and CKO mice were normalized to tibia length (n = 6-7). (F) Quantification of cardiomyocyte cross sectional area in β2AR F/F and CKO hearts (n = 50-60 cells from 5 animals). (G) Detection of mRNA expression of hypertrophic markers Anp, Bnp, and β-Mhc in 8-month-old β2AR F/F and CKO hearts (n = 5). Data are shown in dot plots with mean ± SEM. P values were obtained by one-way ANOVA and Student’s t-test. *P < 0.05; **P < 0.01; ***P < 0.001; ****P < 0.0001.

To evaluate the pathological effects of elevated Ca^2+^/CaMKII activity and CTGF expression on the heart, we performed echocardiography to assess cardiac function over an aging course. Compared to β_2_AR-F/F mice, both systolic and diastolic functions were significantly impaired in β_2_AR-CKO mice, beginning around 4–6 months of age, with further deterioration as they aged (Figure 4A). Cardiac fibrosis was markedly increased in 8-month-old β_2_AR-CKO hearts, evidenced by significantly elevated collagen deposition in the myocardium and upregulation of the expression of fibrotic marker genes, including *Col1a1, Col3a1, Ctgf, Postn*, and *Tgf β1, 2, 3* (Figure 4B–D). Although the ratio of heart weight to body weight was similar between β2AR-CKO and β2AR-F/F, cardiomyocyte hypertrophy was observed in β2AR-CKO hearts, as demonstrated by significantly increased cardiomyocyte cross sectional area and mRNA expression of hypertrophic marker genes, including Anp, Bnp, and β-Mhc (Figure 4E–G). Since expression of Cre recombinase under the control of αMHC promoter has been reported to induce cardiac fibrosis in mice at 6 months of age,^16–18^ we compared cardiac function and fibrosis in β_2_AR-CKO mice with the age-matched αMHC-Cre controls. Although αMHC-Cre mice did not exhibit significant impairment in cardiac function (Figure 4A), collagen deposition in their hearts was notably increased compared to β2AR-F/F hearts, which was significantly lower than that observed in β2AR-CKO hearts (Figure 4B and C). Meanwhile, while literature points to a critical role of β_2_AR-Gi-Akt signaling to protect against myocyte apoptosis,^10^ we did not detect a reduction of Akt activity and an increase in apoptosis in 2- and 8-month-old β_2_AR CKO hearts (Supplementary Figure 1A–C).

### Nifedipine Treatment Improves Cardiac Function and Reduces Fibrosis in β_2_AR-CKO Mice

**Figure 5.**
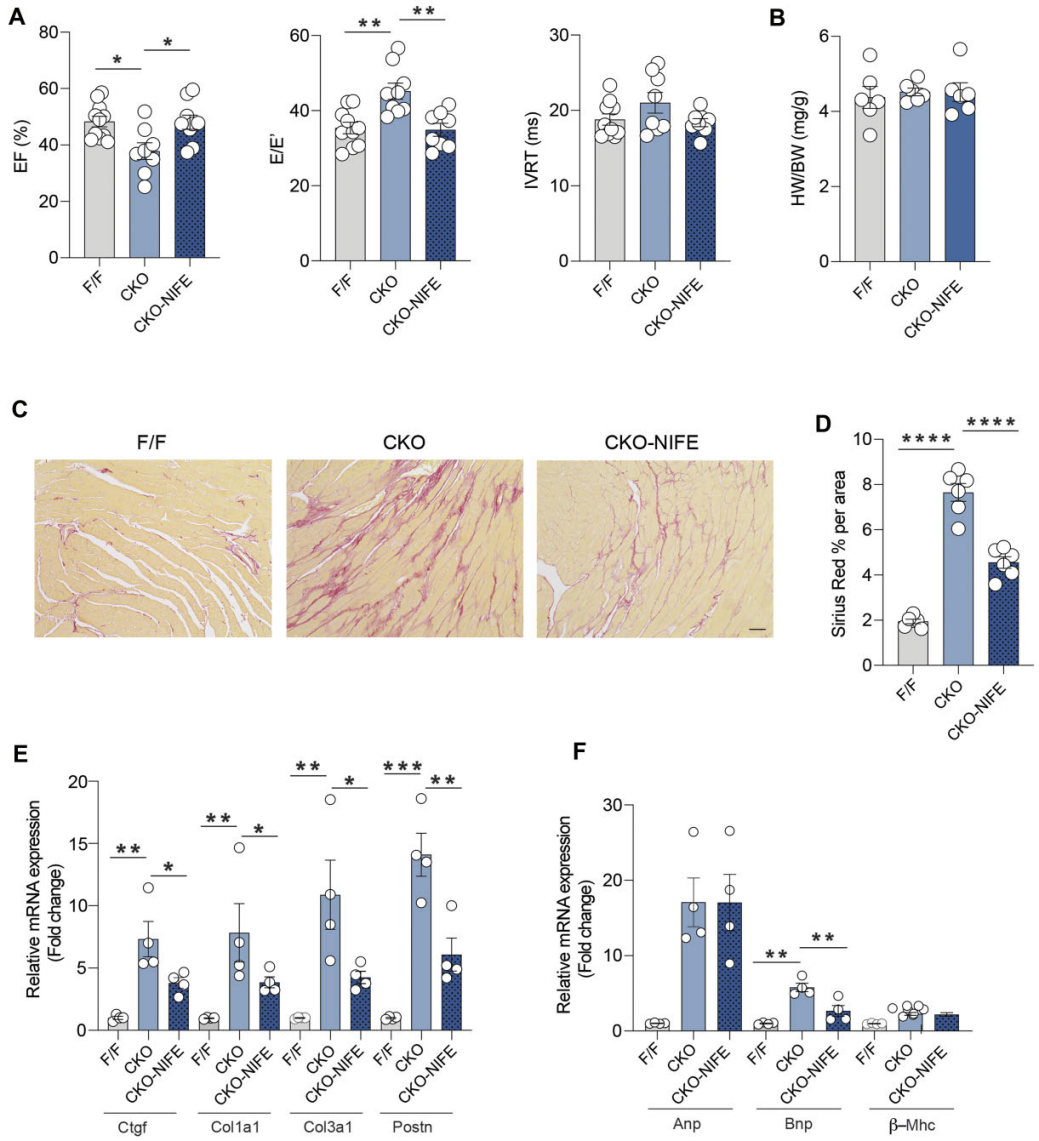
(A) Cardiac function was assessed after 1-month of nifedipine treatment in 6-month-old β_2_AR F/F and CKO mice as EF, the ratio of early diastolic transmitral blood flow velocity (E wave) to early diastolic mitral annular tissue velocity (E’ wave) (E/E’), and IVRT (*n* = 8–10). (B) Heart weights were normalized to body weights in β_2_AR F/F and CKO heart after vehicle or nifedipine treatment (*n* = 6). (C) Representative Sirius Red stains of β_2_AR F/F and CKO heart cross sections after either vehicle or nifedipine treatment (*n* = 6). Scale bar: 50 μm. (D) Quantification of fibrosis (%) by Sirius staining (*n* = 6). (E) mRNA expression of fibrotic genes Ctgf, Col1a1, Col3a1, Postn, and hypertrophic genes Anp, Bnp, and β-Mhc in β_2_AR F/F and CKO heart hearts after either vehicle or nifedipine treatment (*n* = 4). Data are shown in dot plots with mean ± SEM. *P* values were obtained by one-way ANOVA and Student’s *t*-test. *P < 0.05; **P < 0.01; ***P < 0.001; ^****^P < 0.0001.

β_2_AR-CKO and β_2_AR-F/F mice at 6-month-old age were then used for in vivo nifedipine treatment for one month. β_2_AR-CKO mice were subjected to either nifedipine or vehicle treatment, while β_2_AR-F/F mice received vehicle treatment. Cardiac function was assessed via echocardiography. In β_2_AR-CKO mice, nifedipine treatment improved both systolic and diastolic function, as demonstrated by improvements in EF and E/E’ compared to β_2_AR-CKO vehicle group (Figure 5A). Heart weight to body weight ratio and cardiac morphology remained unchanged (Figure 5B). However, cardiac fibrosis was significantly reduced by nifedipine treatment in β_2_AR-CKO hearts (Figure 5C and D). Consistently, the mRNA expression of fibrotic marker genes, including *Col1a1, Col3a1, Ctgf*, and *Postn*, were significantly decreased in β_2_AR-CKO hearts following nifedipine treatment (Figure 5E). While Bnp mRNA expression was reduced, the expression of the other two hypertrophic marker genes, Anp and β-Mhc, remained unchanged (Figure 5F). Overall, these results demonstrate that nifedipine effectively improves cardiac function in β_2_AR-CKO mice by reducing fibrotic gene expression and thereby mitigating fibrosis in the heart.

## Discussion

Our study demonstrated the critical role of cardiomyocyte β_2_AR in preserving Ca^2+^ homeostasis and mediating fibrosis in the heart. Loss of β_2_AR leads to enhanced LTCC phosphorylation and current and CaMKII activity in cardiomyocytes, which induces fibrotic protein CTGF expression in the heart. As a result, an age-dependent cardiac dysfunction accompanied by fibrosis was developed in β_2_AR-CKO hearts, while treatment with the LTCC blocker alleviated the pathological changes. These findings identify an indispensable role of cardiomyocyte β_2_AR in the homeostatic regulation of LTCC and safeguarding the LTCC-dependent Ca^2+^/CaMKII signaling and fibrosis in the heart.

Initial activation of CaMKII requires elevated intracellular Ca^2+^.^19^ The level of intracellular Ca^2+^ depends on both the Ca^2+^ cycling between the cytosol and the extracellular space, as well as between the cytosol and intracellular Ca^2+^ stores. Therefore, Ca^2+^ channels located at both the plasma membrane and intracellular organelles are essential regulators of the cytosolic Ca^2+^ signaling. Our data indicate that an elevation of LTCC underlies the increased intracellular Ca^2+^ and CaMKII activity in β_2_AR-CKO myocytes. Meanwhile, a sustained and excessive CaMKII activation is also considered to be an upstream signaling for increased LTCC opening probability.^20,21^ In β_2_AR-CKO hearts, the LTCC blocker, nifedipine, completely attenuated the CaMKII activity, indicating a positive feedback loop between CaMKII and LTCC in response to the sympathetic tone in β_2_AR-CKO hearts. While our data support that the changes of LTCC activity contribute to the Ca^2+^-CaMKII activation in β_2_AR-CKO cardiomyocytes, we cannot completely rule out the potential contribution of Ca^2+^ from the intracellular Ca^2+^ stores and the indirect impacts of the drug on vasculature.

In the heart, while Ca^2+^ plays a central role in controlling ECC, it also influences diverse signaling in regulating gene expression. Disruptions in intracellular Ca^2+^ signaling-dependent gene expression are inevitably involved in the progression of maladaptive cardiac remodeling.^2^ CaMKII is one of the critical downstream effectors that mediates maladaptive cardiac remodeling.^19^ Deletion of CaMKII abrogates cardiac inflammation, fibrosis, and dysfunction without affecting hypertrophy in a mouse disease model induced by transaortic constriction.^22^ CTGF is an important molecule mediating fibrosis in many cell types. CTGF is upregulated in both cardiomyocytes and cardiac fibroblasts in the failing heart^23^ and myocardial injured heart.^24^ It has been demonstrated that stimulations with phenylephrine and endothelin promote upregulation of CTGF mRNA and protein in cardiomyocytes, but not in cardiac fibroblasts.^25^ Our data show the profibrotic CTGF is upregulated in the young β_2_AR-CKO cardiomyocytes and hearts, which was attenuated by LTCC and CaMKII inhibitors. These data support that CTGF is downstream of Ca^2+^-CaMKII activation in β_2_AR-CKO cardiomyocytes. Therefore, the initial upregulation of CTGF in cardiomyocytes, probably acting as a paracrine factor, contributes to increased cardiac fibrosis in β_2_AR-CKO hearts. In the pathogenesis of myocardial fibrosis, evidence also supports that transforming growth factor-β (TGF-β) acts as a profibrotic cytokine to drive CTGF expression.^26^ However, we didn’t detect a significant change of TGF-β expression in the young β_2_AR-CKO heart, indicating that CTGF acts upstream of TGF-β in this model, and supporting a feedback loop between CTGF and TGF-β to promote cardiac fibrosis.

In cardiomyocytes, stimulation of cardiac β_1_AR drives cAMP-PKA mediated activation of LTCC and intracellular Ca^2+^ signaling.^27^ In comparison, stimulation of cardiac β_2_AR has been implicated in coupling to Gi and phosphodiesterases to limit cAMP-PKA and LTCC activity and Ca^2+^ signaling.^14^ We detected increased phosphorylation of LTCC in β_2_AR CKO hearts. In isolated β_2_AR CKO cardiomyocytes, *I_Ca_* was significantly increased under the stimulation of isoproterenol, indicating a sustained alteration of Ca^2+^ signaling due to the alteration of βAR signaling under the sympathetic tone. Interestingly, the antagonism between β_2_AR and β_1_AR and other GPCRs are recently extended from the plasma membrane to the intracellular compartments in cardiomyocytes. β_1_AR has been identified to be located in the Golgi apparatus and activates phospholipase C (PLC) at the Golgi apparatus and subsequent phosphorylates protein kinase D and histone deacetylase 5 (HDAC) driving pathological hypertrophy.^28^ It has been recently shown that internalized β_2_AR signaling inhibits β_1_AR-mediated PLCε activation at the Golgi apparatus,^29^ consistent with the baseline cardiac hypertrophic phenotype observed in β_2_AR-CKO hearts. Accordingly, the loss of β_2_AR could enhances CaMKII and hypertrophic signals induced by β_1_AR- or other neurohormones, exacerbating cardiac dysfunction in cardiomyopathy induced by high fat diet feeding and myocardial infarction.^11^

While literature points to a critical role of β_2_AR-Gi-Akt signaling to protect against myocyte apoptosis,^10^ we did not detect reduction of Akt activity and increase in apoptosis in β_2_AR CKO hearts. Therefore, at the baseline, the β_2_AR-Akt pathway has limited role in the observed pathology in CKO hearts. In contrary, our data uncover the critical role of cardiac β_2_AR signaling in preventing chronic activation of CaMKII by maintaining Ca^2+^ homeostasis. These findings provide a mechanism supporting the cardiomyocyte-specific β_2_AR-dependent protection against the development of cardiac fibrosis and hypertrophy.

## Supplementary Material

zqaf036_Supplementary_material_20250803

## Data Availability

The datasets supporting the conclusions of this article are included within the article and its supplemental files.
