# Supplementary material for: Cardiac β2 adrenergic receptor deletion drives calmodulin kinase II upregulation to induce connective tissue growth factor in cardiac fibrosis and diastolic dysfunction
Source: Function (Oxf). 2025 Aug 12;6(5):zqaf036. doi: 10.1093/function/zqaf036 (PMC12448417; doi:10.1093/function/zqaf036)
Supplement: zqaf036_Supplementary_material_20250803 [file zqaf036_supplementary_material_20250803.pdf]

Table S1. Real-time PCR primers

Primer	Sequence (5’ - 3’)
Ctgf - Forward	CTTCTGCGATTTCGGCTCC
Ctgf - Reverse	TACACCGACCCACCGAAGA
Tgf β1 - Forward	ACCAAAGACATCTCACACAG
Tgf β1- Reverse	GTAACGCCAGGAATTGTTGC
Tgf β2 - Forward	CATCTCCTGCTAATGTTGTTG
Tgf β2 - Reverse	GGCGAAGGCAGCAATTATC
Tgf β3 - Forward	CACAGAGCAGAGAATTGAGC
Tgf β3 - Reverse	CAGTGACATCGAAAGACAGC
Postn - Forward	TGCTGCCCTGGCTATATGAG
Postn - Reverse	GTAGTGGCTCCCACAATGCC
Col1a1 - Forward	TAGGCCATTGTGTATGCAGC
Col1a1 - Reverse	ACATGTTCAGCTTTGTGGACC
Col3a1 - Forward	TATAAGCCCTGATGGTTCTC
Col3a1 - Reverse	CATCTTGCAGCCTTGGTTAG
α-Sma - Forward	AAGAGCATCCGACACTGCTGAC
α-Sma - Reverse	AGCACAGCCTGAATAGCCACATAC
Gapdh - Forward	CATGGCCTTCCGTGTTCCTA
Gapdh - Reverse	CCTGCTTCACCACCTTCTTGAT
Anp - Forward	TCGTCTTGGCCTTTTGGCT
Anp - Reverse	TCCAGGTGGGCTAGCAGGTTCT
Bnp - Forward	CTCCTGAAGGTGCTGTCC
Bnp - Reverse	GCCATTTCCTCCGACTTT
β-Mhc - Forward	ATGTGCCGGACCTGGGAAG
β-Mhc - Reverse	CCTCGGGTTAGCTGAGAGATCA
β_2AR - Forward	CTGGTTGGGCTACGTCAACT
β_2AR - Reverse	TCCGTTCTGCCGTTGCTATT

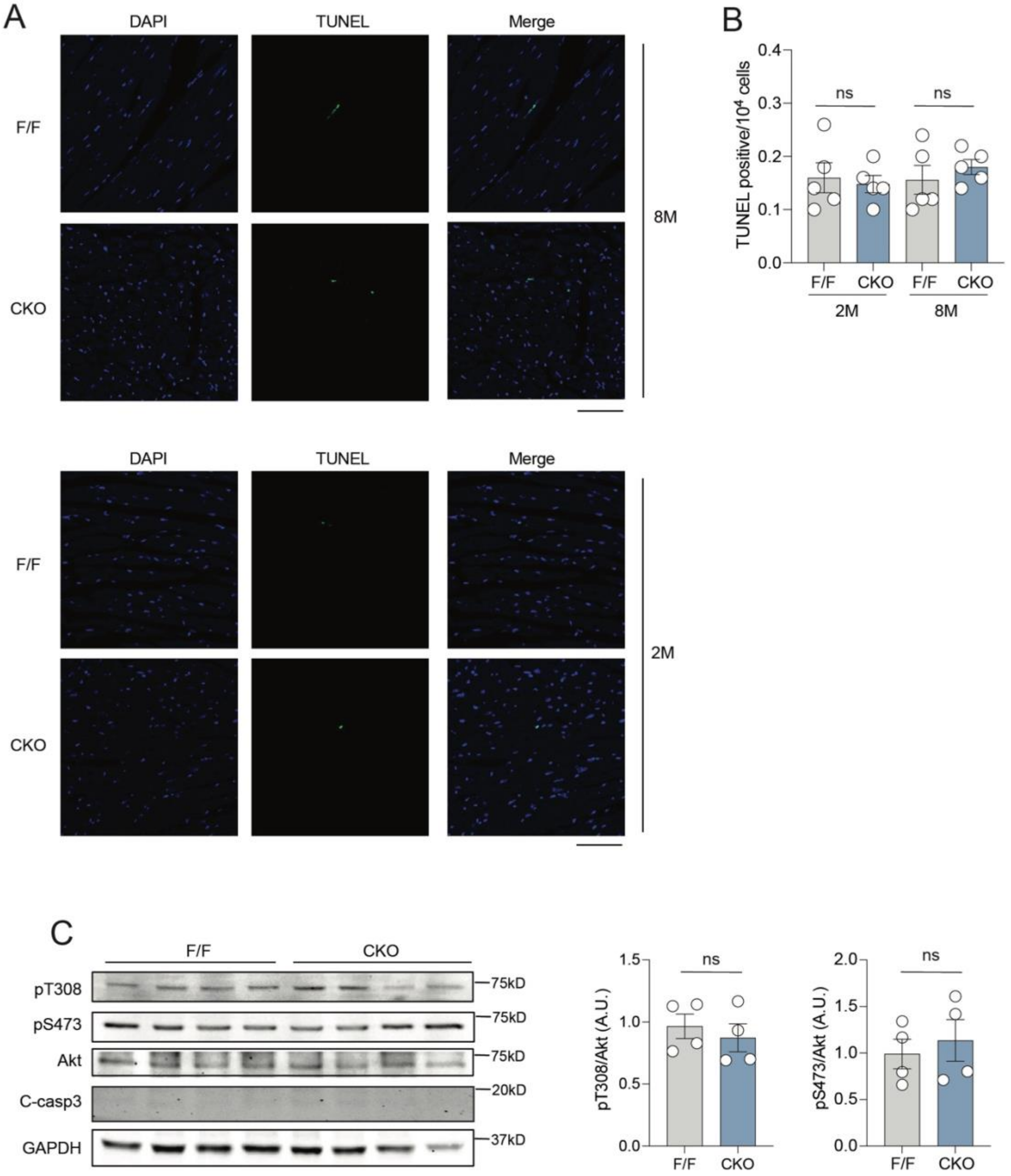
A
DAPI
TUNEL
Merge
F/F
CKO
8M
2M
B
TUNEL positive/10⁴ cells
0.0
0.1
0.2
0.3
0.4
ns
F/F
CKO
2M
8M
C
F/F
CKO
pT308
pS473
Akt
C-casp3
GAPDH
75kD
20kD
37kD
pT308/Akt (A.U.)
0.0
0.5
1.0
1.5
pS473/Akt (A.U.)
2.0

Figure S1. Cardiomyocyte deletion of β_2AR does not affect cell apoptosis and AKT activity in the heart. (A) Representative images show TUNEL staining in 2-month-old and 8-month-old β_2AR F/F and CKO heart sections. Nuclei are shown with DAPI staining. Scale bar: 75 μm. (B) Quantification of TUNEL staining (n = 5). (C) Representative immunoblots of phosphorylated AKT, total AKT, cleaved-caspase 3 (C-casp 3) in 2-month-old β_2AR F/F and CKO heart tissues and quantification as indicated (n = 4). 2M: 2-month; 8M: 8-month. A.U. indicates arbitrary unit. Data are shown in dot plots with mean ± SEM. P values were obtained by two-way ANOVA and Student's t-test. ns: not significant.
